# Linalool-Incorporated Synergistically Engineered Modified Liposomal Nanocarriers for Enhanced Transungual Delivery of Terbinafine against Onychomycosis

**DOI:** 10.3390/ma16124424

**Published:** 2023-06-16

**Authors:** Isha Gupta, Syeda Nashvia Adin, Md Abdur Rashid, Yahya Alhamhoom, Mohd. Aqil, Mohd. Mujeeb

**Affiliations:** 1 Phytomedicine Laboratory, Department of Pharmacognosy & Phytochemistry, School of Pharmaceutical Education & Research, Jamia Hamdard, New Delhi 110062, India; 2 Department of Pharmaceutics, College of Pharmacy, King Khalid University, Al Faraa, Abha 62223, Saudi Arabia; 3 Department of Pharmaceutics, School of Pharmaceutical Education & Research, Jamia Hamdard, New Delhi 110062, India

**Keywords:** terbinafine, invasomes, onychomycosis, Box–Behnken design, anti-fungal study

## Abstract

This work investigates the synthesis of linalool-containing invasomes for terbinafine (TBF-IN) in order to increase the solubility, bioavailability, and nail permeability of terbinafine (TBF) for transungual administration. TBF-IN was created utilising the thin-film hydration technique, and with the Box–Behnken design (BBD), optimisation was carried out. TBF-INopt were investigated for vesicle size, zeta potential, PDI (Polydispersity index), entrapment efficiency (EE) and in vitro TBF release. In addition, nail permeation analysis, TEM (transmission electron microscopy), and CLSM (confocal scanning laser microscopy) were performed for further evaluation. The TBF-INopt exhibited spherical as well as sealed vesicles with a considerably small size of 146.3 nm, an EE of 74.23 per cent, a PDI of 0.1612, and an in vitro release of 85.32 per cent. The CLSM investigation revealed that the new formulation had better TBF nail penetration than the TBF suspension gel. The antifungal investigation demonstrated that the TBF-IN gel has superior antifungal activity against *Trichophyton rubrum* and *Candida albicans* compared to the commercially available terbinafine gel. In addition, an investigation of skin irritation using Wistar albino rats indicates that the TBF-IN formulation is safe for topical treatment. This study confirmed that the invasomal vesicle formulation is an effective vehicle for the transungual delivery of TBF for the treatment of onychomycosis.

## 1. Introduction

Onychomycosis is among the most prevalent nail diseases that infest the nail plate and nail bed. It is the most prevalent fungal ailment that is ascribable to non-dermatophyte moulds (*Scopularicpsis brevicaulis*, *S. dimidiatum*, *Scytalidium hyalinum*, *Aspergillus* sp., and *Acremonium* sp.), dermatophytes (*Trichophyton rubrum*, *Epidermophyton floccosum*, *T. mentagrophytes*, and *T. krajdenii*), and yeasts (*Candida albicans* and other candida species) [1]. Recent studies have revealed that Trichophyton mentagrophytes and Trichophyton rubrum account for 90% of onychomycosis [2]. It usually manifests as onycholysis, subungual hyperkeratosis, nail plate thickening, nail discolouration, and loss of nail plate consistency as a prodrome eventuating in paraesthesia and local pain [3]. Disease progression augments with contemporaneous inhabitation of other diseases such as diabetes, HIV, psoriasis, immunosuppression, peripheral vascular disease, tinea pedis, and trauma [1].

The treatment of onychomycosis becomes notoriously strenuous owing to various factors viz. high cost, rigid nail structure, high chances of relapse, and prolonged treatment duration [4].

Oral therapeutics, due to their accessibility and efficacy, are extensively used for the primary treatment of onychomycosis. The medications used are azoles (fluconazole and itraconazole) and allylamines (terbinafine) [5,6,7]. Howbeit, their effectiveness is constrained attributable to their restricted availability at the active site, which further enhances the treatment cost and duration [3]. Hepatotoxicity and cardiac disturbances are major snags associated with oral therapeutics. In such a scenario, topical delivery of drugs is merely tempting as it will increase the concentration at the desired site, circumvent the drawbacks associated with the oral route, and thus revamp the therapeutic efficacy [8].

Terbinafine, a synthetic allylamine drug, is used orally for the treatment of systemic and superficial fungal infections attributable to its high efficacy and broad spectrum of activity against non-dermatophytes, candida species, and dermatophytes [9]. The topical ungual delivery of terbinafine is a tempting approach that will circumvent the limitation associated with the parenteral and oral routes. Howbeit, the formidable keratinous structure of the nail circumvents the permeation of the drug to the deep-seated infection. Henceforth, to triumph over this barrier, various tactics have been used, amongst which the use of penetration enhancers excels over other approaches. Permeation enhancers disrupt the dense keratinous nail plate and thus augment the diffusion gradient and permeation of the drug through the nail plate [10,11,12,13,14,15,16,17,18,19,20].

Invasomes are flexible and novel vesicular carriers comprising a blend of lipid, terpene, and ethanol with more flair for transungual delivery of drugs than liposomes (structure is shown in Supplementary Materials) [21].

Our previous research unveiled that linalool (a natural terpene) can be used as a potential permeation enhancer to augment the transungual delivery of terbinafine [22,23]. Henceforth, our present study is aimed to develop a nano-invasomal formulation comprising terbinafine along with linalool, which is a penetration enhancer, using the thin-film hydration method to augment the terbinafine transungual delivery. BBD was utilised for the developed formulation optimisation with phospholipon 90G, ethanol, and linalool serving as independent variables whose effects were evaluated with dependent variables such as vesicle size, EE, and PDI. In addition, the optimised preparation (TBF-INopt) was cast into a carbapol-934 P-based gel and examined for CLSM, nail penetration research, in vitro release kinetics, and antifungal efficacy against *Trichophyton rubrum* and *Candida albicans*.

## 2. Materials and Methods

“Terbinafine standard and Linalool were obtained from Sigma Aldrich (Karnataka, India)”, “Terbinafine active pharmaceutical ingredient was obtained from Virupaksha Organics Limited (Telangana, India)”, and “triethanolamine obtained from Fischer Scientific (Mumbai, India)”. “Lipoid (Ludwigshafen am Rhein, Germany) supplied Phospholipon 90G”, while “SD fine chemicals supplied Polyethylene glycol-400, Methanol, Chloroform, and carbapol 934P. (Mumbai, India)”. “SD Fine chemicals was the source of all HPLC solvents (Mumbai, India)”.

### 2.1. Terbinafine Loaded Invasomes (TBF-IN) Formulation Preparation

Using the thin film hydration approach, terbinafine-loaded invasomes were produced. In a round-bottom flask (RBF), chloroform: methanol (1:3, *v*/*v*) was used to dissolve a set amount of phospholipon 90G (lipid), terbinafine (drug-1 mg/mL), and linalool. The organic solvent was evaporated with a rotary evaporator under vacuum to form a thin coating of lipid on the RBF walls. The round-bottomed flask was stored overnight in a desiccator. Using a 7:3 ratio of water to ethanol, the dried film was rehydrated for one hour and refrigerated to achieve appropriate expansion. The obtained mixture was probe sonicated for four min with a “titanium probe ultra sonicator” (“UP 100 H, Hielscher Ultrasonics GmbH, Berlin, Germany”) [24].

### 2.2. Optimisation of Invasomes Using BBD

Primary screening studies were conducted to identify the characteristics that may influence the potential benefits of invasomes for transungual administration. Design expert version 13 software was used to generate a three-factor BBD after identifying acceptable qualities (“State-ease, Minneapolis, USA”). BBD was used to determine the influence of linalool, lipid, and ethanol content on the response variables such as EE vesicle size and PDI. The design included seventeen experimental runs. The Box–Behnken design yielded a quadratic response surface model that was as follows:

Z = k_0_ + k_1_X_1_ + k_2_X_2_ + k_3_X_3_ + k_12_X_1_X_2_ + k_13_X_1_X_3_ + k_23_X_2_X_3_ + k_11_X_12_ + k_22_X_22_ + k_33_X_32_

Herein, Z indicates “Predicted response”;
X_i_ indicates “independent variables”;
The variables k_i_, k_i2_, and k_i3_ indicate “quadratic, linear as well as interactive coefficients”.

The adopted “independent factors” were the concentrations of linalool (X_3_), ethanol (X_2_), and phospholipon 90G (X_1_), while the “dependent variables” were the “PDI (Y_1_), EE (Y_3_) and vesicle size (Y_2_)” (shown in Table 1).

### 2.3. Characterisation of TBF-INpt

#### 2.3.1. Vesicle Size, Zeta Potential, and PDI

The zeta potential, PDI, and vesicle size of the prepared TBF-IN were determined with dynamic light scattering (DLS) using a “zetasizer (Malvern instruments, Wancestershine, UK)” at 25 ± 1 °C in triplicate following 100-fold dilution of the formulation with Mill-Q water at a 90 °C scattering angle [25].

#### 2.3.2. EE

Ultracentrifugation was used to determine the TBF-IN EE [24]. The samples (1 mL) were kept overnight at 4 °C and then centrifuged at 20,000 rpm for 1 h at 4 °C using a “REMI cooling centrifuge (Mumbai, India)”. The filtrate comprising free terbinafine was removed and assessed for terbinafine content using HPLC after diluting the formulation with the appropriate medium. The EE was examined with the following formula:

$$\%\;\mathrm{EE}=\frac{Total\;terbinafine-Terbinafine\;in\;supernatant\times 100}{Total\;terbinafine}$$

The terbinafine concentration was determined using HPLC with C_18_ column and mobile phase acetonitrile: methanol: triethylamine (35:55:10, % *v*/*v*) at 1 mL per min flow rate. The UV detection was performed at 280 nm [22].

### 2.4. Invasomes Morphology

TEM was utilised to perform a morphological investigation of the TBF-INopt (“TEM-Tecnai, CM 200, Philips scientific, New York, NY, USA”). On a copper grid, a drop of diluted material was used prior to analysis, which was followed by a TEM investigation after negative staining with 2 per cent phosphotungstic acid [26].

### 2.5. Formulation of Terbinafine-Loaded Invasomal Gel

To prolong the formulation’s retention on the nail, TBF-INopt was converted into a transungual gel. The gel was created by dissolving a fixed quantity of “carbapol-934 P” (one per cent *w*/*w*) in distilled water and allowing it to expand overnight. Subsequently, after adding “triethanolamine” (for pH-alteration), “15 percent *w*/*w* polyethylene glycol 400” (as a plasticizer), and “chlorocresol” (0.1 percent as a preservative), the optimised terbinafine-loaded invasome was added dropwise with continuous homogenization to obtain a homogeneous gel combination [27].

### 2.6. pH and Texture Analysis of TBF-INopt Gel

The “TA-XT plus texture analyzer” (“Stable Micro system, Godalming, UK”) was used to perform the TBF-INopt gel texture analysis based on several criteria including “hardness”, “adhesiveness”, “work of cohesion”, and “cohesiveness”. The research was done by putting TBF-INopt gel in a glass beaker and compressing it (50 mL). The probe travelled a distance of 10,000 mm at a “test speed of 2.0 mm/s”. The probe was brought back using the “post-test speed of 2.0 mm/s”. On contact with the gel, the upper probe met the automatic “trigger force of 10.0 g”. The programme “Texture Exponent 32” was utilised to analyse the force necessary to separate the probe from the gel. The pH was measured using a digital pH metre after dissolving one gram of gel in 100 millilitres of distilled water to determine the pH (“Eutech pH 700 Eutech Instruments, Singapore”) [28].

### 2.7. In Vitro TBF Release Study

The “in-vitro release” of TBF suspension gel (control) and TBF-INopt gel was determined using a drug release dialysis membrane approach. Both formulations (1 mg/g) were loaded onto a 12,000–14,000 Da “preactivated dialysis membrane” (Hi Media, Mumbai) that was attached to the shafts of a 500 mL beaker comprising phosphate buffer saline (pH 6.8) with uniform stirring at 37 ± 2 °C and 100 rpm. At predetermined intervals of 30, 60, 120, 240, 480, 720, and 1440 min, samples were taken and supplemented with new release media. The terbinafine concentration was determined using the RP-HPLC technique, and a graph was drawn between time (hours) and cumulatively drug release percentage [29,30].

### 2.8. Nail Permeation Study

The Franz-diffusion cell was used to evaluate the nail penetration of TBF-INopt and TBF suspension gel (control). A “Franz-diffusion cell with a 2.0 cm^2^ effective permeation area and a 15 mL receiving cell capacity” was utilised. The “goat hooves” were purchased from a butcher shop, cleaned, rinsed with phosphate buffer saline, and attached to the receiver compartment. The recipient cell was thronged with the release medium, while the donor cell was thronged with TBF-INopt gel. The steady temperature was maintained for 24 h at 37 ± 2 °C with continual stirring at 150 rpm. At predefined time intervals of 30, 60, 120, 240, 480, 720, and 1440 min, samples were collected and replaced with new release medium, and the terbinafine concentration was determined using the RP-HPLC technique [24].

### 2.9. CLSM

The TBF solution containing “rhodamine B dye” (control-1 mL) and “rhodamine B dye-loaded invasomal formulation” (1 mL) were used to excise goat hooves for 72 hrs at 37 degrees Celsius and then mounted on “Franz diffusion cells”. After 72 h, samples of “goat hooves were cleaned with distilled water to eliminate excess dye. The samples were then placed on glass slides and chopped into thin slices with a thickness of 6–10 μm. The slide was then examined using “CLSM (Leica TC SPE-1lw, Leica microsystem, Wetzlar, Germany)” with an “argon laser beam (excitation at 488 nm and emission at 570 nm)” [24].

### 2.10. Skin Irritation Investigation

The Draize score test was used to assess skin irritation caused by the TBF-INopt gel using rats. Nine Wistar albino rats were divided into three groups of three animals each to evaluate the skin irritancy of the TBF-INopt gel: Group 1 was administered a 10% formalin solution, Group 2 was administered the TBF-INopt gel, and Group 3 was administered a commercially available terbinafine gel (Terbinaforce 1 per cent). The untreated vicinal region on the rats was utilised as the control skin, and erythema and oedema scores were calculated. The formulation was applied after removing hair from the skin of the rats, and following sample removal, erythema and oedema were evaluated visually [31].

### 2.11. Assessment of Antifungal Activity

The fungal strain of *Candida albicans* and *Trichophyton rubrum* were spread over yeast potato dextrose agar (YPDA) media in Petri plates, respectively, and then the TBF-IN gel (1%) and TBF-marketed gel (Terbinaforce 1%) were placed aseptically in the respective marked places on the plates. Further, the plates were kept in the refrigerator for 1 h at 4 °C, and then plates were incubated in incubator for 24 h at 28 °C temperature. After 24 h, the plates were measured for zone of inhibition in mm [31].

#### Statistical Analysis

The data were displayed as “mean ± standard deviation of the mean”. The collected data were analysed using the “Dunnett *t* test” and a “one-way analysis of variance”.

## 3. Results and Discussion

Nanoparticle-based topical drug delivery is advantageous due to the nanoparticles’ tiny size and direct administration at the target region [32]. Vesicular nanoparticles enhance the colloidal stability of nanoparticles, lessen nanoparticle aggregation, and modify drug release [33]. Invasomal nanoparticles were created and integrated into a gel in this work to examine their potential for transungual TBN delivery.

The prime consideration that determines whether a topical therapy for nail disorders is effective is the formulation’s capability to transport adequate quantities of medicine into and across the nail membrane. In this instance, the use of potent chemical permeation enhancers may boost the transfer of drugs through the nail plate. Therefore, selecting the most effective enhancers is essential for maximizing medication absorption in nail plates [34]. To assess numerous compounds and find the effective agent, our previous study used a high throughput technique, which is typically used in screening nail permeation enhancers [35]. The effect of the most common ungual permeability-enhancing drugs on the build-up of terbinafine in nail tissue was examined. In terms of drug accumulation in the nail tissue, the results indicated that linalool performed much better than the other chemicals evaluated in this study. The potential of linalool to increase water absorption and swelling in the nail plate, which finally softens and reduces barrier resistance while increasing nail plate permeability, may be the underlying mechanism [36]. Additionally, increased water absorption in the nail causes the keratin to be more hydrated, which improves the diffusion of medication molecules [36].

### 3.1. Optimisation of TBF-IN Formulation with BBD

**Figure 1:**
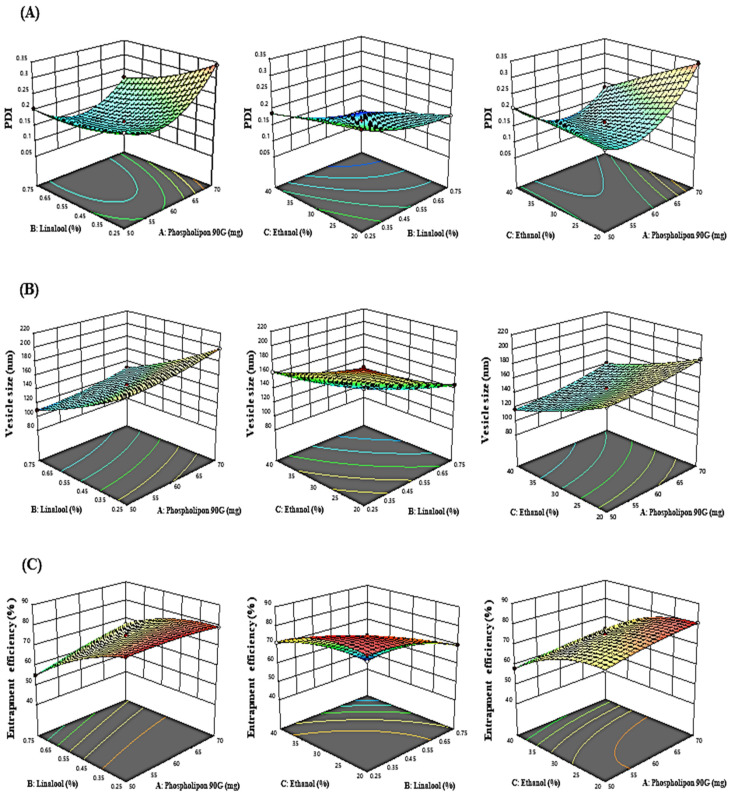
Three-dimensional response graphs illustrating the effect of the independent variables on the (**A**) PDI, (**B**) vesicle size, and (**C**) entrapment efficiency.

**Figure 2:**
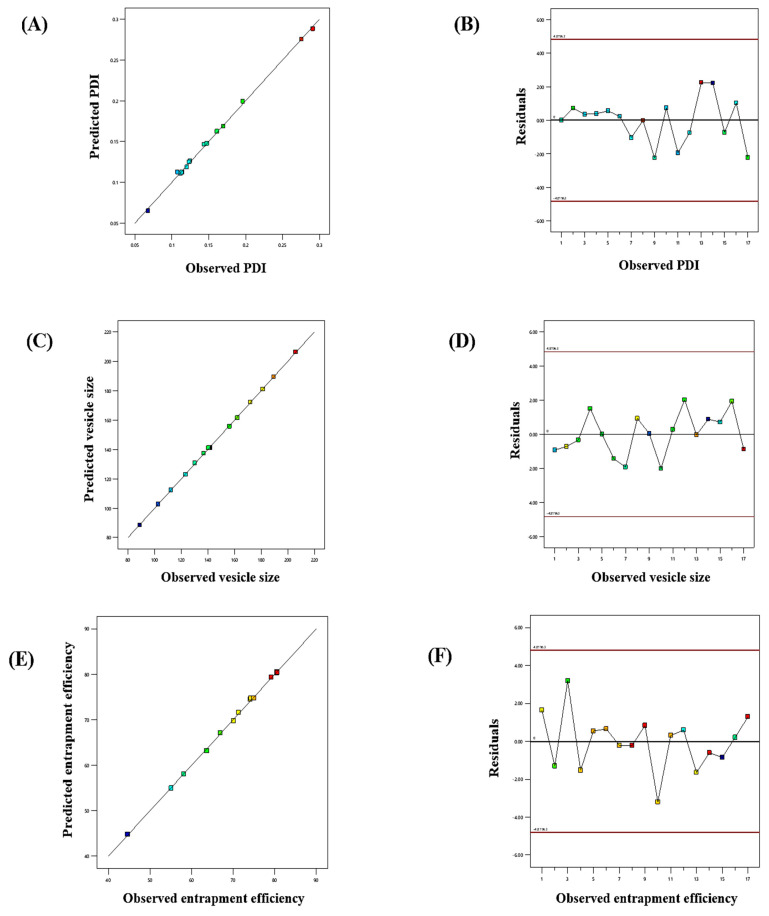
Linear correlation plots (**A**,**C**,**E**) between the actual and predicted values and the corresponding residual plots (**B**,**D**,**F**) for various responses.

The influence of the “adopted parameters (ethanol, Phospholipon 90G, and linalool) on PDI, EE, and vesicle size” on the TBF-loaded invasomes is illustrated in the three-dimensional response diagram shown in Figure 1, and the “corresponding residual plots for adopted responses and linear correlation between experimental and predicted values” (generated with BBD) are shown in Figure 2.

#### 3.1.1. Response (1): Effect of Independent Variables on PDI

The PDI of all 17 runs were estimated to be between 0.0926 and 0.3425 (Table 2).

PDI = +0.1611 + 0.0320A − 0.378B − 0.0348C − 0.0227AB − 0.0441AC + 0.0081BC + 0.0728A^2^ + 0.0165 B^2^ − 0.0024 C^2^

Phospholipon 90G had a favourable influence on PDI, as shown in the polynomial equation above. On raising the phospholipon 90G (50–70 mg) concentration, the PDI enlarged from 0.2325 ± 0.003 to 0.3408 ± 0.003, 0.2054 ± 0.009 to 0.2229 ± 0.004, 0.1856 ± 0.005 to 0.2086 ± 0.007, and 0.1892 ± 0.008 to 0.3425 ± 0.009 as noted in formulations 2 and 17, 3 and 15, 1 and 7, and 16 and 4, respectively. However, ethanol and linalool have a negative impact on PDI. Similarly, raising the ethanol concentration (20 to 40%) caused a reduction in PDI from 0.1793 ± 0.004 to 0.0926 ± 0.009, 0.2416 ± 0.003 to 0.1873 ± 0.002, 0.1892 ± 0.008 to 0.2086 ± 0.004, and 0.3425 ± 0.009 to 0.1856 ± 0.007 as noted in formulations 12 and 11, 13 and 14, 16 and 7, and 4 and 1, respectively. Similarly, raising the linalool concentration from 0.25 to 0.5% led to a decrease in PDI from 0.2325 ± 0.003 to 0.2054 ± 0.009, 0.1873 ± 0.002 to 0.0926 ± 0.009, 0.3408 ± 0.003 to 0.2229 ± 0.007, and 0.2416 ± 0.003 to 0.1793 ± 0.004 as noted in formulations 2 and 3, 14 and 11, 17 and 15, and 13 and 12, respectively.

#### 3.1.2. Response (2): Effect of Independent Variables on Vesicle Size

The vesicle size of all 17 runs was estimated to be between 93.82 and 212.29 (Table 2).

Vesicle size = +146.10 + 10.58 A − 33.79B − 24.78C − 0.5000AB + 1.57AC − 0.8300BC + 3.53A^2^ + 3.92B^2^ + 3.86C^2^

According to experimental findings, phospholipon 90G had a favourable influence on the size of vesicles. On raising the concentration of phospholipon 90G from (50–70 mg), the vesicle size increased from 176.07 ± 3.92 to 198.15 ± 3.92 nm, 109.96 ± 2.98 to 130.04 ± 2.32 nm, 116.96 ± 4.03 to 141.35 ± 2.32 nm, and 168.78 ± 3.07 to 186.89 ± 2.98 nm as noted in formulations 2 and 17, 3 and 15, 7 and 1, and 16 and 4, respectively. This might be explained by the fact that increasing phospholipon 90G causes the bilayer width to expand, which in turn causes a noticeable rise in vesicle size. In contrast, ethanol and linalool had an adverse effect on the size of the vesicles. On raising the concentration of ethanol (20 to 40 per cent), the size of the vesicles decreased from 145.91 ± 4.23 to 93.82 ± 2.98 nm, 212.29 ± 3.92 to 163.52 ± 2.09 nm, 168.78 ± 3.07 to 116.96 ± 4.03 nm, and 186.89 ± 2.98 to 141.35 ± 4.09 nm as noted in formulations 12 and 11, 13 and 14, 16 and 7, and 4 and 1, respectively. Similarly, on raising the linalool concentration from (0.25 to 0.75%), the size of the vesicles decreased from 176.07 ± 3.92 to 109.96 ± 2.98 nm, 163.52 ± 2.09 to 93.82 ± 2.98 nm, 198.15 ± 3.92 to 130.04 ± 2.32 nm, and 212.29 ± 3.92 to 145.91 ± 4.23 nm as noted in formulations 2 and 3, 14 and 11, 17 and 15, and 13 and 12, respectively. The reduction in the vesicle size induced by linalool and ethanol may be a result of the rupture in the bilayer structure of the cellular membrane when their concentrations are exceeded.

#### 3.1.3. Response (3): Effect of Independent Variables on EE

According to experimental findings, it was shown that the independent factors had a substantial impact on EE and that the EE of all 17 runs ranged between 44.62 and 80.62% (Table 2).

EE = +74.75 + 2.80A − 9.36B − 8.46C + 3.28AB − 0.2325AC − 4.05BC − 0.9435A^2^ − 3.38B^2^ − 4.71C^2^

Based on above-mentioned polynomial equation, it was determined that phospholipon 90G had a positive impact on EE, whereas ethanol and linalool had a negative impact. It was discovered that a rise in the concentration of phospholipon 90G (50 to 70 mg) lead to an increase in EE from 79.18 ± 1.68 to 80.54 ± 1.68%, 55.12 ± 2.09 to 66.89 ± 1.23%, 58.13 ± 2.98 to 63.65 ± 1.34%, and 74.09 ± 2.09 to 80.54 ± 2.09% as noted in formulations 17 and 2, 3 and 15, 7 and 1, and 16 and 4, respectively. This may be due to the increase in the bilayer domain dimension caused by the creation of a greater number of invasomal vesicles, which allows more space for TBF entrapment in IN vesicles.

According to the experimental data, the EE of TBF in IN vesicles decreased as the ethanol concentration (20 to 40%) rose from 70.12 ± 1.62 to 44.62 ± 2.09%, 80.62 ± 1.68 to 71.32 ± 1.76%, 74.09 ± 2.09 to 58.13 ± 2.98%, and 80.54 ± 2.09 to 63.65 ± 1.34% as noted in formulations 12 and 11, 13 and 14, 16 and 7, and 4 and 1, respectively. Similarly, a rise in the linalool concentration (0.25 to 0.75%) caused a reduction in the entrapment efficiency from 80.54 ± 1.68 to 55.12 ± 2.09%, 71.32 ± 1.76 to 44.62 ± 2.09%, 79.18 ± 1.68 to 66.89 ± 1.23%, and 80.62 ± 1.68 to 70.12 ± 1.62% as noted in formulations 2 and 3, 14 and 11, 17 and 15, and 13 and 12, respectively. This may be because ethanol and linalool alter the bilayer membrane structure of vesicles over a specific concentration, which causes drug loss from invasomal vesicles.

On the basis of the above-mentioned experimental findings, an optimised formulation containing ethanol (30%), phospholipon 90G (60 mg), and linalool (0.5%) established in accordance with the formula generated with the “point prediction method” and evaluated for PDI, EE, and vesicle size. The TBF-INopt displayed a vesicle dimension of 146.3 ± 3.92 nm, an EE value of 74.23 ± 1.68%, and a PDI value of 0.1612 ± 0.003, which were close to the Box–Behnken anticipated values for PDI of 0.1611, vesicle dimension of 146.10 nm, and EE of 74.75%.

### 3.2. Characterisation

**Figure 3:**
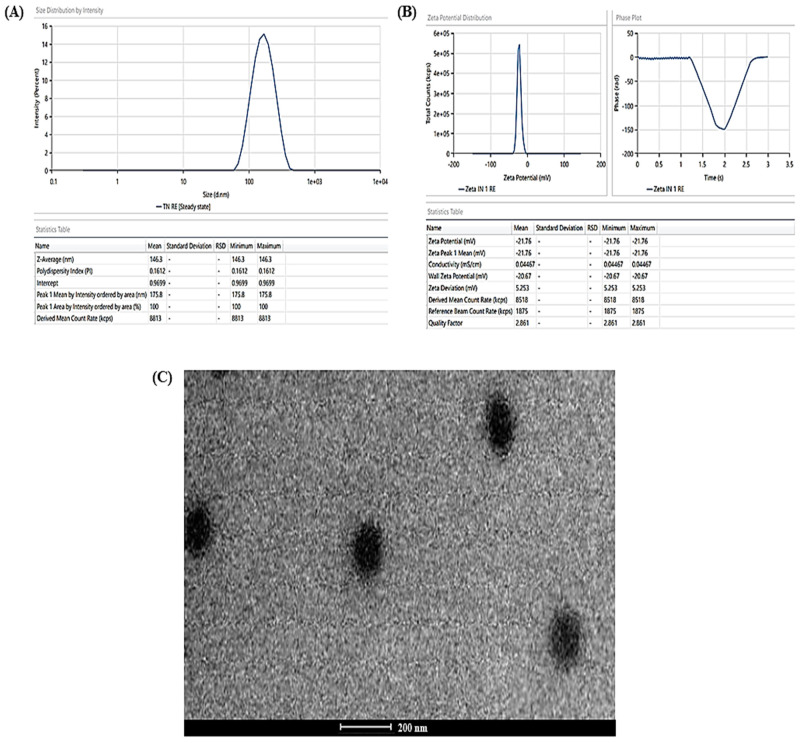
(**A**) Vesicle size distribution in the TBF-INopt formulation, (**B**) zeta-potential of the TBF-INopt formulation, and (**C**) transmission electron microscopy showing the TBF-INopt formulation.

The experimentally determined average vesicle size and PDI of TBF-INopt were 146.3 nm and 0.1612, respectively (Figure 3A), with an EE of 74.23%, while the anticipated values were 146.10 nm and 0.1611, with an EE of 74.75%. The calculated values of all responses were in close proximity to their expected values, supporting the validity and consistency of the model. In addition, the TBF-INopt zeta potential was estimated to be −21.76 mV. (Figure 3B).

### 3.3. Invasomes Morphology

The TEM analysis of the TBF-INopt formulation indicated that the resulting vesicles had a circular morphology, a well-defined, firmly packed structure, and a consistent size distribution (illustrated in Figure 3C).

The TEM analysis might also assist with a number of other crucial issues, including the visual assessment of particulate size and distribution, the existence of drug crystals independent of nanovesicles, and particle aggregation [37].

The pictures revealed discrete spheroids particles with sizes falling within the range specified using the “DLS particle size analysis”. It should be emphasised that the variances in particle size as assessed with TEM and DLS are to be anticipated due to differences in the preparation of sample techniques [38]. DLS assessed the suspension hydrodynamic radius, whereas TEM determined the particle size. The photos of the optimised nanovesicles lacked evidence of aggregated particles or drug crystals. In addition, it was essential to determine the particle shape because there is evidence in the literature to the opposite, although this may have been the result of unique experimental circumstances in previous research [39]. Vesicular nanoparticles have been studied as improved drug delivery mechanisms for decades, and topical cutaneous and ungual novel antifungal formulations have recently attracted the curiosity of researchers [40]. Nanocarrier-based topical treatments have distinct advantages, such as focused medication delivery, that traditional approaches cannot match. Elsherif et al. [41] developed nano-vesicular terbinafine formulation, and Naumann et al. [42] developed new antifungal liposomal formulations. These are recent examples illustrating the topical formulation’s continuous release, which is crucial because these treatments frequently necessitate long-term maintenance [43]. One of the most essential characteristics of these formulations is their tiny particle size, which is helpful for permeation and may aid in increased drug deposition in the surface layers [44]. The deposition of drug-containing particles in tissue layers, cavities, and folds might result in long-lasting and effective skin and mucosa therapies while minimizing systemic exposure to potentially dangerous medications [45,46].

### 3.4. pH and Texture Analysis of the TBF-INopt Gel

**Figure 4:**
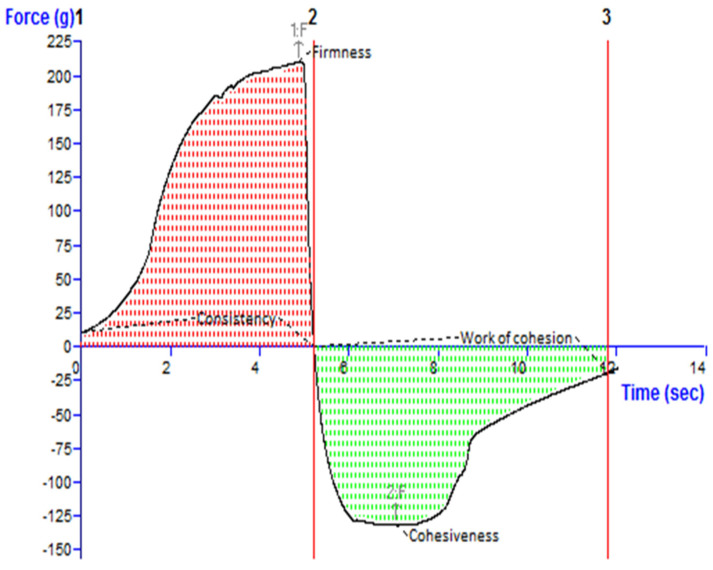
Texture analysis diagram showing the consistency, firmness, work of cohesion, and cohesiveness of the TBF-INopt gel.

Figure 4 depicts an investigation of the texture of the TBF-INopt gel. The “hardness”, “cohesiveness”, consistency, and “work of cohesion” of TBF-INopt were estimated to be 210.17 g, −132.80 g, 681.57 g·s, and −533.49 g·s, respectively (illustrated in Figure 4).

It was established that the pH of the nano-gel was 6.9 ± 0.1. This implies that the gel medication will not cause irritation when it comes into contact with the tissue around the nails.

The literature suggests that the solubility of TBF rises with a lowering pH, which is an additional benefit of a higher pH [47,48]. Additionally, this circumstance may favour the partitioning of the medicine into the vehicle prior to administration as nanovesicles. Terbinafine is ionised at low pH values and binds to keratin in the nail [49]. The ionised terbinafine increases in molecular weight and becomes less permeable when it is released from the nanovesicles and dissolved in the hydrogel. In addition, the nail plate, consisting of keratins with disulphide bonds, has a net negative charge at physiological pH (pH 7.4) and an isoelectric point (pI) between 4.0 and 5.0 [25,50], generating favourable circumstances for negatively charged nanovesicles to stick to the nail, even in deeper layers owing to their small particle size, and resulting in a more effective drug delivery mechanism.

### 3.5. In Vitro Drug Release Study

**Figure 5:**
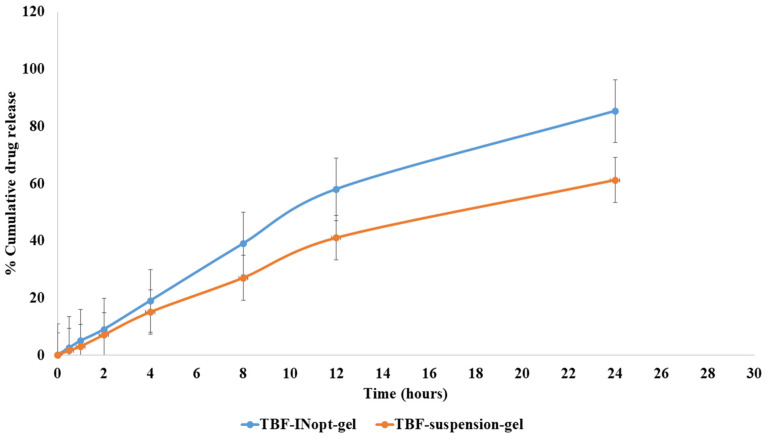
Comparative in vitro drug release profile for the TBF suspension and TBF-INopt formulation.

The in vitro release of terbinafine from a TBF solution across a dialysis membrane was anticipated to be 61.23 per cent; however, the optimised TBF-INopt formulation demonstrated an 85.32 per cent release of terbinafine over a “dialysis membrane” (Figure 5). At each time point, a large amount of the medication was released. The TBF-INopt formulation displayed a delayed drug release compared to pure TBF. IN is capable of controlling drug release due to the fact that TBF must penetrate the lipid bilayer and can diffuse slowly. The graph depicts the rapid release of the medication over the first four hours, followed by a gradual release over the next twenty-four hours. This type of releasing behaviour is good for enhancing the efficacy of therapy. An initial quick release assists in establishing a therapeutic concentration, while a longer slow release promotes therapeutic efficacy [24]. “Different mathematical kinetics models (korsmeyer peppas, zero-order, first-order and Higuchi kinetics model)” were used to fit in vitro drug release experiment data, with the “Higuchi kinetics model” yielding the highest R^2^ value, as shown in Table 3. The release of terbinafine from the TBF-INopt gel therefore follows a “higuchi diffusion process”.

### 3.6. Nail Permeation Study

**Figure 6:**
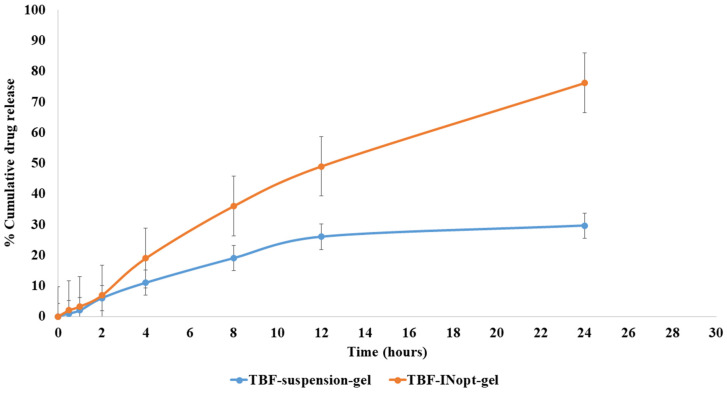
Comparative in vitro nail permeation study of the TBF suspension and TBF-INopt formulation across goat hooves.

The nail permeation investigation demonstrated that 29.62% of terbinafine permeated through the TBF suspension gel, whereas 76.21% of terbinafine permeated through the TBF-INopt formulation (Figure 6). The addition of ethanol and terpene facilitates TBF solubilisation and results in 2.57-fold more TBF penetration from TBF-INopt nanovesicles than the TBF suspension [24].

### 3.7. CLSM

**Figure 7:**
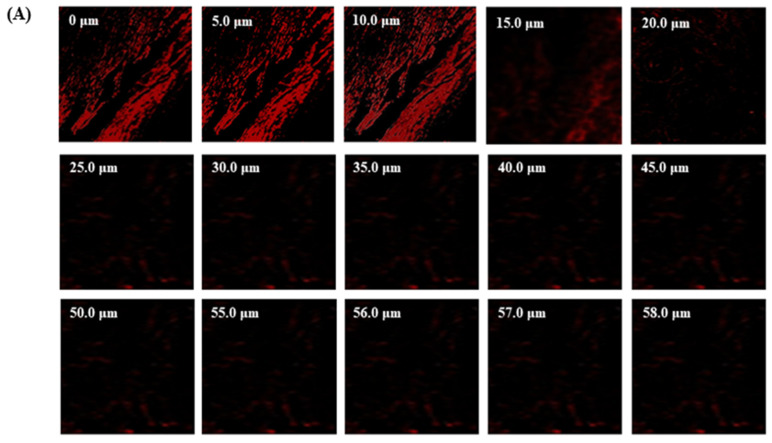

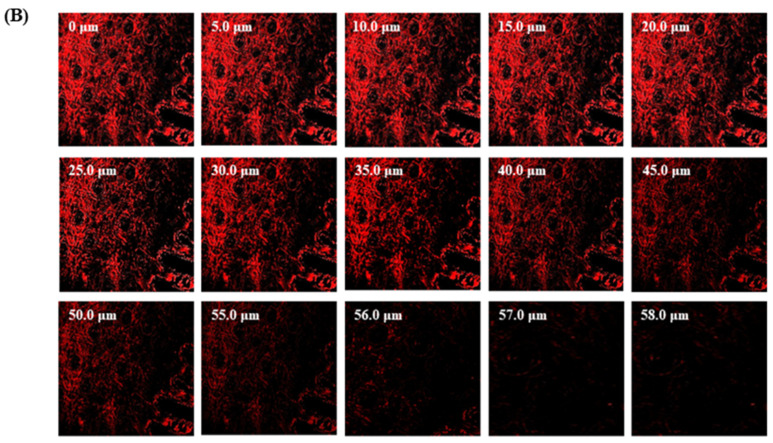
CLSM images in an optical cross-section perpendicular to the goat hooves (**A**) treated with the TBF suspension and (**B**) treated with the TBF-INopt formulation.

The TBF-IN formulation entered the keratinous layer of the goat hooves (up to 56 μm) deeper than the TBF solution (control), which was restricted to 15 μm per se (Figure 7). The TBF-INopt formulation’s increased fluorescence intensity revealed that TBF was evenly dispersed throughout the greater depths of the goat hooves to a greater extent than the TBF suspension, confirming the enhanced permeation.

### 3.8. Skin Irritation Investigation

**Figure 8:**
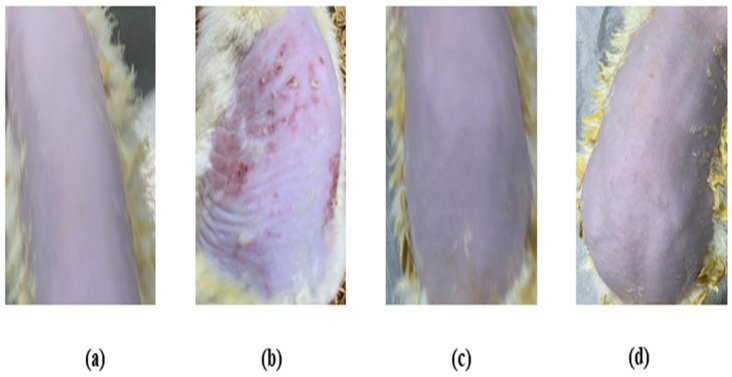
Skin irritation images of rats treated with the (**a**) normal control, (**b**) positive control, (**c**) TBF-INopt gel, and (**d**) terbinafine-marketed gel.

The skin irritation investigation of the TBF-IN gel was carried out using Wistar albino rats (Figure 8) and evaluated by comparing the groups treated with the formalin solution and conventionally marketed terbinafine gel. The erythema and oedema scores for the treatment groups are reported in Table 4. The group treated with the formalin solution demonstrated a high irritation scale for oedema (2.67 ± 0.31) and erythema (3.33 ± 0.42), but the group treated with the TBF-IN gel exhibited an extremely low irritation scale for oedema (0.33 ± 0.02) and erythema (0.0 ± 0.00). The group treated with the marketed terbinafine gel exhibited little irritation with an irritation scale for oedema (0.33 ± 0.02) and erythema (0.33 ± 0.02). The group treated with the TBF-IN gel lacked irritation and redness. Based on the above findings, it can be concluded that the TBF-IN gel formulation is non-irritant.

### 3.9. Antifungal Activity of the TBF Invasomal Gel

**Figure 9:**
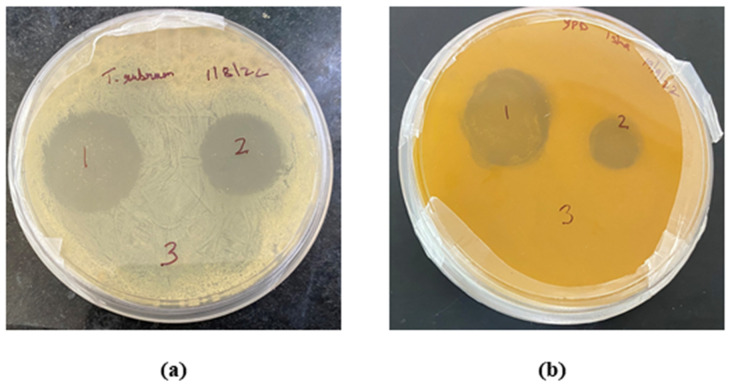
Plates showing the zone of inhibition for the (1) TBF-INopt gel, (2) terbinafine-marketed gel, and (3) control against (**a**) *Trichophyton rubrum* and (**b**) *Candida albicans*.

The in vitro antifungal activity depicted with the zone of inhibition produced by the TBF-IN gel (1%) and terbinafine-marketed gel (1%) were estimated to be 30 mm and 19 mm against *Trichophyton rubrum* and 28 mm and 12 mm against *Candida albicans*, respectively. The improved antifungal activity of the TBF-IN gel can be attributable to enhanced diffusion of TBF-containing invasomal vesicles through the fungal cell walls resulting in more inhibition of ergosterol synthesis [24]. Hence, the TBF-IN gel exhibited greater inhibitory activity in comparison with the terbinafine-marketed gel (Figure 9). To confirm that the antifungal activity is not due to the presence of other formulation components of invasomes, an antifungal study was also conducted using vehicle control. The results of the zone of inhibition for the control formulation were found to be zero, which confirms the antifungal activity of the terbinafine-loaded invasomal gel. The results (Table 5) show that the terbinafine-loaded invasomal gel possesses potent antifungal activity.

## 4. Conclusions

This study focused on the development and optimisation of terbinafine-containing invasomal formulations using BBD, which resulted in improved antifungal effectiveness against onychomycosis when administered transungually. The TBF-IN formulation exhibited a nano size of 146.3 nm, an EE of 74.23%, and a PDI of 0.1612. The CLSM analysis revealed that the new TBF-IN formulation had better TBF nail penetration than the TBF suspension gel, as well as a larger in vitro release. The antifungal investigation revealed that the TBF-IN formulation was more effective against Trichophyton rubrum and Candida albicans than the regular terbinafine gel on the market. The results demonstrate that the produced invasomal vesicle formulation is a valuable vehicle for the transungual administration of TBF for the treatment of onychomycosis.

## Figures and Tables

**Table 1:** Variables and their levels.

Variables	Levels		
	Low (−1)	Medium (0)	High (+1)
Independent Variables			
A: Phospholipon 90G (mg)	50	60	70
B: Linalool (%)	0.25	0.5	0.75
C: Ethanol (%)	20	30	40
Dependent Variable			
Y_1_: PDI Y_2_: Vesicle size (nm) Y_3_: Entrapment efficiency (%)			

**Table 2:** BBD experimental design with measured responses.

Formulation	Independent Variables			Dependent Variables		
	A	B	C	Y_1_	Y_2_	Y_3_
1	70	0.5	40	0.1856 ± 0.007	141.35 ± 4.09	63.65 ± 1.34
2	50	0.25	30	0.2325 ± 0.003	176.07 ± 3.92	80.54 ± 1.68
3	50	0.75	30	0.2054 ± 0.009	109.96 ± 2.98	55.12 ± 2.09
4	70	0.5	20	0.3425 ± 0.009	186.89 ± 2.98	80.54 ± 2.09
5	60	0.5	30	0.1612 ± 0.003	146.3 ± 3.92	74.23 ± 1.68
6	60	0.5	30	0.1634 ± 0.003	145.8 ± 3.92	74.97 ± 1.68
7	50	0.5	40	0.2086 ± 0.003	116.96 ± 4.03	58.13 ± 2.98
8	60	0.5	30	0.1602 ± 0.012	146.2 ± 4.26	75.01 ± 1.62
9	60	0.5	30	0.1599 ± 0.005	145.7 ± 4.36	74.67 ± 1.72
10	60	0.5	30	0.1608 ± 0.003	146.5 ± 4.67	74.88 ± 1.87
11	60	0.75	40	0.0926 ± 0.009	93.82 ± 2.98	44.62 ± 2.09
12	60	0.75	20	0.1793 ± 0.004	145.91 ± 4.23	70.12 ± 1.62
13	60	0.25	20	0.2416 ± 0.003	212.29 ± 3.92	80.62 ± 1.68
14	60	0.25	40	0.1873 ± 0.002	163.52 ± 2.09	71.32 ± 1.76
15	70	0.75	30	0.2229 ± 0.004	130.04 ± 2.32	66.89 ± 1.23
16	50	0.5	20	0.1892 ± 0.008	168.78 ± 3.07	74.09 ± 2.09
17	70	0.25	30	0.3408 ± 0.003	198.15 ± 3.92	79.18 ± 1.68
**Quadratic model**		**R^2^**	**Adjusted R^2^**	**Predicted R^2^**	**S.D.**	**%CV**
**Response (Y_1_)**		0.9995	0.9989	0.9943	0.0021	1.02
**Response (Y_2_)**		0.9998	0.9996	0.9979	0.5902	0.3897
**Response (Y_3_)**		0.9992	0.9983	0.9915	0.4178	0.5925

**Table 3:** In vitro drug release kinetics of different models with their correlation values.

Release Kinetics	R^2^	Equation	*X*-Axis	*Y*-Axis
Korsmeyer–Peppas	0.976	M_t_/M∞ = K_tn_	Log fraction of drug released	Log time
Higuchi	0.989	M_t_ = M_0_ + k_h_t_1/2_	Fraction of drug released	√time
Zero-order release	0.951	M_t_ = M_0_ + k_0_ t	Fraction of drug released	time
First-order release	0.966	ln M_t_ = ln M_0_ + k_1_ t	Log% drug remaining	time

**Table 4:** Draize irritation score after application of the TBF-IN gel, TBF-marketed gel, and formalin solution on Wistar albino rats.

Rat	Positive Control		TBF-IN Gel		TBF-Marketed Gel	
	Edema	Erythema	Edema	Erythema	Edema	Erythema
1	2	4	0	0	0	1
2	3	3	1	0	1	0
3	3	3	0	0	0	0
Mean ± SD	2.67 ±0.31	3.33 ±0.42	0.33 ±0.02	0 ± 0	0.33 ±0.02	0.33 ± 0.02

**Table 5:** Zone of inhibition (in mm) for the (1) TBF-INopt gel, (2) terbinafine-marketed gel, and (3) control against *Trichophyton rubrum* and *Candida albicans*.

Zone of Inhibition (in mm)				
S. No.	Microbe	Control Vehicle	TBF-IN Gel	TBF-Marketed Gel
1	*Trichophyton rubrum*	0 ± 0	30 ± 2	19 ± 3
2	*Candida albicans*	0 ± 0	28 ± 2	12 ± 2

## Data Availability

This study did not report any data (all the data are included in the current manuscript).

## Supplementary Materials

The following supporting information can be downloaded at: https://www.mdpi.com/article/10.3390/ma16124424/s1. Reference [51] is cited in the supplementary materials.
