# Supplementary material for: Linalool-Incorporated Synergistically Engineered Modified Liposomal Nanocarriers for Enhanced Transungual Delivery of Terbinafine against Onychomycosis
Source: Materials (Basel). 2023 Jun 16;16(12):4424. doi: 10.3390/ma16124424 (PMC10301328; doi:10.3390/ma16124424)
Supplement: Supplementary file 1 [file materials-16-04424-s001.zip › materials-2345222-supplementary.pdf]

Supporting information:

Mechanism of invasome formulation (TBF-IN) :

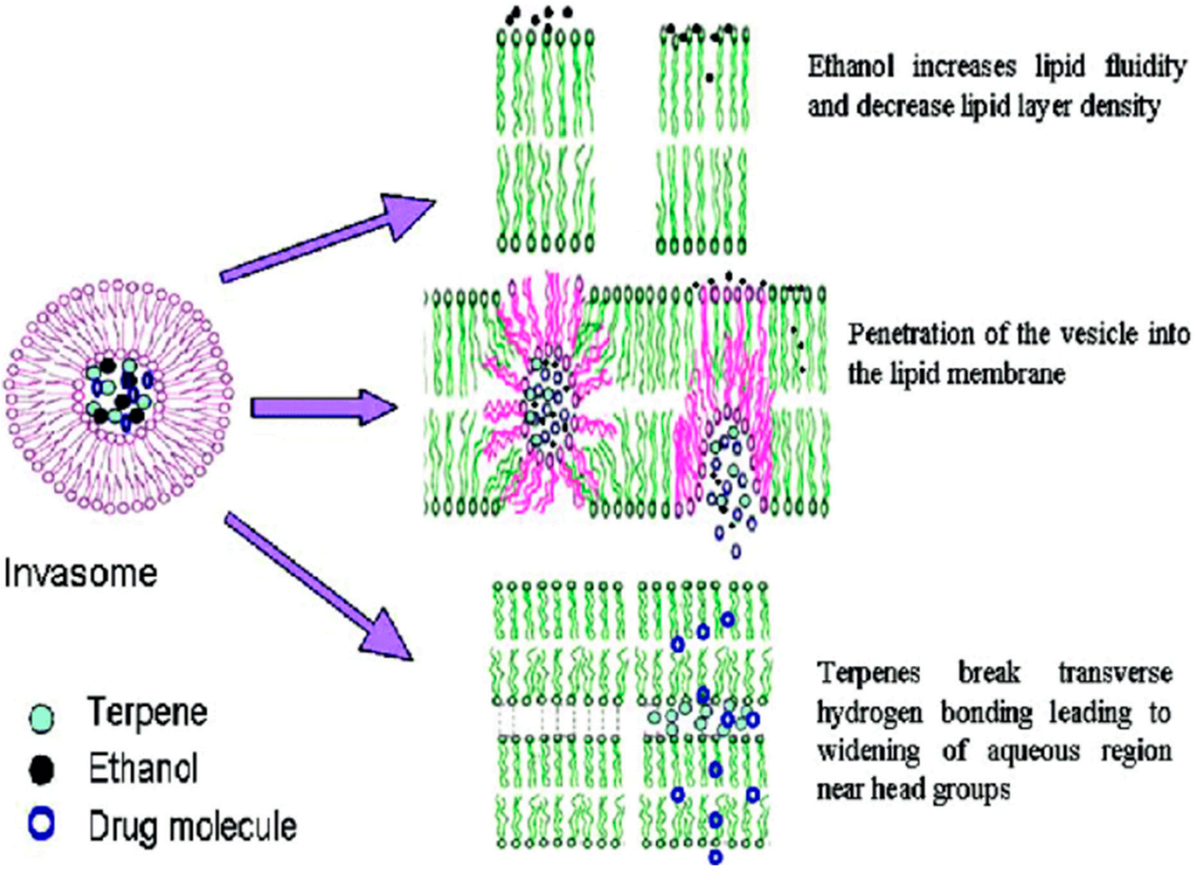

Ref:

Lakshmi, PK & Kalpana, B & Domaraju, Prasanthi. (2013). Invasomes-novel Vesicular Carriers for Enhanced Skin Permeation. Systematic Reviews in Pharmacy. 4. 26. 10.4103/0975-8453.135837.
